# Ecological Momentary Assessment in Patients With an Acquired Brain Injury: A Pilot Study on Compliance and Fluctuations

**DOI:** 10.3389/fneur.2020.00115

**Published:** 2020-03-05

**Authors:** Saskia D. Forster, Siegfried Gauggel, Axel Petershofer, Volker Völzke, Verena Mainz

**Affiliations:** ^1^Institute of Medical Psychology and Medical Sociology, University Hospital of the RWTH Aachen, Aachen, Germany; ^2^VAMED Klinik Hattingen GmbH, Rehabilitation Centre for Neurology, Neurosurgery, Neuropaediatrics, Hattingen, Germany

**Keywords:** ecological momentary assessment, EMA, experience sampling method, ESM, acquired brain injury, ABI, neuropsychological assessment, feasibility

## Abstract

Ecological Momentary Assessment (EMA) promises to be a suitable method for capturing the dynamics in self-assessments through repeated measurements in naturalistic environments using common mobile devices. Therefore, EMA could increase the power of neuropsychological assessment by obtaining a more fine-grained picture of symptoms, limitations, and strengths in patients with an acquired brain injury (ABI) in real-life situations. The present study examined 15 patients with an ABI with cognitive and motor impairments. Following a semirandomized high-frequency sampling plan to assess EMA's feasibility and applicability, data were collected across 7 days. At eight prompts per day, patients were asked about their current activities, the social context they were in, their current mood, performance judgments of their own functional status, and the frequency of self-reflections. The average compliance rate was 71.6%. The fluctuations in patients' responses were measured in terms of variance distributions within simple (intercept only) three-level models and root mean square of successive difference values. They were sufficient, as shown, for example, by the mean within-person variability of 44.9% across all of the items studied. There were no significant correlations between patients' age, severity of depressive symptoms, or their level of functioning and their compliance with study participation or the variability of their responses. The results support the feasibility and applicability of EMA as an assessment technique in patients with an ABI. There are, however, limitations that should be considered when planning an assessment of brain-injured patients using EMA.

## Introduction

An acquired brain injury (ABI) results in complex and multifaceted impairments of sensory–motor, cognitive, emotional, and behavioral functions (1). As a result of these impairments, serious activity limitations and participation restrictions frequently occur (2), and these dramatically change both the patient's life and the life of their family members.

Clinical neuropsychologists have developed sophisticated and elaborate assessment techniques for investigating the multiple behavioral, cognitive, and emotional consequences of an ABI (3). As a result of the rapid advances in technology (e.g., virtual reality, smartphones), various technologies are also beginning to be used to improve the efficiency, reliability, and cost-effectiveness of neuropsychological assessments (4). The Ecological Momentary Assessment (EMA), which is also called the Experience Sampling Method, is such a new technique (5). The EMA is a structured diary technique for assessing patients' subjective experiences in their daily life by employing commonly used mobile devices. The EMA makes use of time-triggered signals or specific events to initiate data collection. It has been applied in patients with somatic illnesses (6) and patients with mental disorders (e.g., mood disorders) (7) to assess their experiences and behaviors and also the momentary context (e.g., location, company) in which the patient is currently involved. Some of the reasons for the popularity of this technique include its strong ecological validity; the focus on the patient's current situation-specific experience and therefore the reduction in memory strains; and the ability to assess contexts, fluctuations, and moment-to-moment changes in patients' mental states (5).

Some studies have already demonstrated the feasibility of using EMA in patients with an ABI (8–15). However, four of these studies were based on the same sample that was originally tested (8, 14, 15) or a subsample of it (13) and should therefore not to be considered as an independent measure of feasibility. The published studies, nevertheless, generally support the suitability of using EMA to assess the subjective experiences of patients with an ABI. Thematically, these studies have mainly focused on how patients' mood is related to different everyday situations. Even though these studies support EMA as being suitable for identifying these associations, only one of the studies (11) adhered to a high-frequency sampling plan. Such a sampling strategy has the distinct advantage of being able to record a detailed, representative sample of patients' self-perceived symptoms over the course of an entire day. The precision of the resolution is another important advantage of EMA compared to using a single (retrospective) assessment. There remains, therefore, a need to further elucidate the extent to which EMA is in fact feasible when it is used with a high-frequency sampling strategy, which may be more burdensome for participants than less frequent sampling strategies.

There is another important aspect of EMA, which, to the best of our knowledge, has not yet been investigated in patients with an ABI. It concerns the extent to which patients' responses might fluctuate over the course of the assessment period (e.g., mood changes during a day or week) and whether the degree of the fluctuation and patients' accompanying compliance are related to specific characteristics of the patients. The EMA is recommended particularly for the assessment of constructs that vary across time or are situation-specific, for example, the level of fatigue that patients experience at different times of the day (16–18). Because high-frequency assessments have little advantage over single assessments or questionnaire studies when they are measuring constructs that remain stable across time and in different situations, it is important to identify the degree of response fluctuation that occurs when EMA is being used. There is, in fact, evidence that people's behavior and their inner experiences do fluctuate according to the situation that they are in. Muraven et al. (19), for example, found that social drinkers more likely exceeded their self-imposed drinking limit on days when they were experiencing more self-control demands. There is also evidence that the degree of fluctuation in patients' self-reported symptoms is related to their individual characteristics. Hallensleben et al. (20), e.g., reported that the degree of fluctuation in patients' suicidal ideations appears to be related to the severity of their depressive symptoms.

An important characteristic of patients with an ABI is that they tend to minimize the impact of the ABI on their competence compared to the ratings of both clinicians and family members on both standardized tests and functional tasks (21). Another purpose of the present study was therefore to assess how patients with an ABI would evaluate their own functional status and whether their evaluations would fluctuate across time.

In summary, the objectives of the current study were as follows: (a) to further establish the feasibility of using EMA (i.e., in terms of patients' compliance) in patients with an ABI and (b) to map fluctuations in patients' responses regarding their mood, performance judgments of the functional status, and the frequency of self-reflection. Finally, we wanted to determine whether patients' compliance and their fluctuations in response behavior are related to their level of functioning, such as their level of basic daily functioning and the severity of their cognitive impairment.

## Methods

### Participants

Twenty-nine German-speaking inpatients in a hospital for neurorehabilitation who were diagnosed with different forms of ABI were contacted by their attending neuropsychologist about participating in the study. Patients were invited to participate if they met the study's inclusion and exclusion criteria. These patients were aged between 18 and 70 years, and the level of their cognitive, visual, and motor functioning indicated that they would be able to use a mobile device. Patients with severe problems with memory, executive functions, and motor performance and those whose orientation was distorted or whose language was impaired were not included. Patients were also excluded if they were not scheduled to remain in the hospital for at least another week. All assessments and the EMA were performed in the hospital. The characteristics of the patients who were included in the sample are shown in Table 1.

**Table 1:** Sample description and test results.

**Age (y)**	**Diagnosis**	**Type of Motor Impairment**	**Days since diagnosis**	**Education** **(y)**	**VLMT learning (T)**	**VLMT consolidation (T)**	**VLMT corrected recognition (T)**	**ANT RT (ms)**	**DESC**	**Barthel index**
51–55	Brain infarction (MCA)		788	20	48 (48)	7 (<26)	9 (40)	NA	0	80
46–50	Brain infarction (basal ganglia)	Hemiparesis	24	22	56 (55)	−1 (63**–**66)	15 > 53)	NA	NA	50
18–25	Traumatic brain injury		89	12	63 (60)	3 (50)	NA	490	1	35
61–65	Traumatic brain injury		171	13	40 (39)	2 (50)	11 (49)	703	14	50
66–70	Brain infarction (MCA)	Hemiparesis	32	13	43 (40)	2 (50)	15 (71)	827	1	35
18–25	Traumatic brain injury		40	16	51 (43)	−1 (66)	15 (>53)	474	7	65
56–60	Brain infarction (MCA)		45	8	24 (<34)	0 (55)	0 (<27)	1,165	15	85
18–25	Encephalitis	Hemiparesis	86	13	55 (48)	0 (63)	12 (45)	478	0	50
56–60	Brain infarction (MCA)	Hemiparesis	26	12	27 (<34)	4 (33)	2 (25)	1,015	9	60
61–65	Brain hemorrhage		24	18	35 (36)	2 (50)	10 (47)	750	4	25
56–60	Brain infarction (MCA)	Hemiparesis	43	24	43 (45)	6 (<26)	12 (46)	831	19	95
51–55	Brain infarction (MCA)		19	14	36 (41)	0 (55)	13 (48)	930	5	50
56–60	Brain infarction (MCA)		50	14	33 (39)	3 (37)	12 (46)	671	12	50
56–60	Ruptured brain aneurysm		101	14	29 (<34)	5 (26)	−7 (<27)	727	3	75
56–60	Ruptured brain aneurysm		65	11	32 (37)	5 (26)	9 (40)	1,153	13	40

Age (y) in years is given in ranges to ensure anonymity.

*MCA, middle cerebral artery; education (y), years of education (school and vocational training); VLMT, raw values of the Verbal Learning and Memory Test with T-values in parentheses; ANT, Attention Network Test; DESC, Rasch-based Depression Screening (clinical cutoff: 12); RT (ms), mean response time in milliseconds; NA, not available, i.e., missing data*.

Of the 29 patients who were initially contacted, 25 agreed to participate, but only 15 of them were actually included [51.7% of the patients who had been contacted; 4 females, 11 males; age mean = 50.7 years (standard deviation (SD) = 16.8); range = 18–67 years]. Patients who were not included either were not willing to participate even though they had initially given their consent (*n* = 3) or did not meet the inclusion criteria despite having been screened by a neuropsychologist (*n* = 3). Additionally, one patient was excluded because of an early discharge, and another three patients were excluded because of technical difficulties. All patients who were included in the final sample received verbal and written descriptions of the purpose and procedure of the study. Each participant's written informed consent was obtained. The study was approved by the ethics committee of the Medical Faculty of RWTH Aachen University (Protocol EK306/17) in accordance with the Declaration of Helsinki.

### Measures

#### Depression Screening

All patients completed the Rasch-based Depression Screening (DESC) (22), which is a 10-item, self-report instrument that assesses the severity of depressive symptoms on a five-point Likert scale ranging from 0 (never) to 4 (always). To assess reliability, Cronbach's α was calculated as a measure of internal consistency. With a Cronbach's α of 0.93, the internal consistency of the DESC was excellent. For the subsequent analyses, total scores were calculated. A total score ≥12 indicates a clinically significant severity of depressive symptoms.

#### Verbal Learning and Memory Test

The Verbal Learning and Memory Test (VLMT) is a serial learning task that measures declarative verbal memory, learning performance, long-term encoding, and recognition performance (3). Administration of the German version of the VLMT closely followed the standardized instructions (23, 24). Each patient's performance on the VLMT was measured in terms of total learning (the sum of trials 1–5, with a maximum score of 75), consolidation performance (the number of words forgotten over time, which was calculated as the difference between the number of words recalled on trials 5 and 7), and corrected recognition (the difference between the number of correctly recognized and incorrectly recognized words, with a maximum score of 15).

#### Attention Network Test

The Attention Network Test (ANT) is a computer-based, reaction time task that measures four components of attention: tonic and phasic alertness and spatial and executive attention (25, 26). On this task, the patients were instructed to respond as quickly as possible to directional stimuli (arrows), which were embedded in specific cues or distractors and designed to stimulate the various components of attention. Specifically, by pressing a key as quickly as possible, patients decided in which direction (left or right) an arrow presented to them was pointing. The arrow was presented along with various cues (warning conditions that indicated the appearance or the location of the target) or distractors (arrows that were flanked on either side by lines, which were congruent or incongruent with the directional stimuli). The patients first performed a practice block of 16 trials, which included feedback on the accuracy of their performance, and then they performed three test blocks of 96 trials each without feedback. The focus of the present study was on tonic alertness, which was captured by mean response times (RT) in milliseconds on correctly performed trials.

#### Barthel Index

The Barthel Index (27) is an assessment of how well 10 basic daily functions (e.g., eating, personal hygiene) are performed. The index can range from 0 to 100; a total score of 0 means that the patient is completely dependent on other people for performing basic daily functions, whereas a total score of 100 means that the patient is able to perform each activity independently. Shah et al. (28) suggested that a score of 0–20 indicates total dependence; 21–60, severe dependence; 61–90, moderate dependence; and 91–99, mild dependence. The head nurse in each respective ward determined the Barthel Index at each patient's admission.

#### Ecological Momentary Assessment

The EMAs were conducted using the software movisensXS, App version 1.3.0 (29) and Android smartphones (Motorola Moto G, third generation), which were provided to the patients for the duration of the study. The concrete wording of the EMA questions in German and their translation into English are shown in Supplementary Table 1.

Altogether, 18 questions, which were divided into five subsets, were included in the EMA. Patients were first asked to identify the activity context they were currently in and to respond using one or more of eleven possible response options. Next, they were asked to indicate the social context they were currently in and to respond using one or more of seven response options. Next, patients were asked to indicate their current level of well-being in the EMA adapted version of the Multidimensional Mood Questionnaire (MDMQ) (30). The MDMQ comprised six bipolar items (e.g., tired—awake). For each item, a seven-point Likert scale was used in which 0 (e.g., tired) was at the left end of the scale and 6 (e.g., awake) was at the right end of the scale. Using the guideline of Wilhelm and Schoebi (30), the MDMQ responses were summarized into three basic mood dimensions: energetic arousal (mean of the items *tired—awake, full of energy—without energy*), calmness (mean of the items *agitated—calm, relaxed—tense)*, and valence (mean of the items *content—discontent, unwell—well*). After completing the mood ratings, patients were asked to evaluate their own performance along nine different functions that are typically impaired following an ABI (e.g., memory, functional independence; Table 2). They were asked to judge their performance on each of the nine functions with the following question: “What (school) grade would you give yourself since the last prompt for…?” The rating categories corresponded to those in the German school-grading system, with 1 indicating *very good* and 6 indicating *very poor* performance. Finally, participants were asked to indicate how much they had thought about themselves since the last EMA prompt. They responded on a six-point Likert scale that ranged from 1 (very little) to 6 (a lot). The purpose of these ratings was to obtain a measure of how frequently the patients had self-reflective thoughts since the previous assessment. With the exception of the mood items, the authors formulated all of the items in order to have a measure of patients' metacognitions (i.e., their judgments of their performance, how many self-reflective thoughts they had had).

**Table 2:** Descriptive statistics for the fluctuations in patients' responses for each variable that was assessed (*n* = 15).

**Scales, items**	**SOV patients (%)**	**SOV days (%)**	**SOV assessments (%)**	**M**	**SD**	**rMSSD (M)**	**rMSSD (range)**
**Mood**^**a**^							
Energetic arousal	33.1	5.5	61.4	3.46	0.82	0.93	0.18–2.54
Calmness	54.0	3.7	42.3	3.84	0.73	0.84	0.15–1.69
Valence	60.6	4.3	35.1	3.91	0.69	0.73	0.14–1.45
**Judgments of performance**^**b**^							
Memory	74.7	3.0	22.4	3.02	0.55	0.59	0–1.73
Functional independence	46.7	15.1	38.2	2.69	0.65	0.70	0–1.69
Reliability	47.2	9.8	43.0	2.36	0.58	0.61	0–1.18
Self-confidence	57.8	3.2	39.0	2.65	0.6	0.69	0–1.91
Learning	60.2	7.0	32.8	2.91	0.61	0.70	0–2.07
Understanding problems	67.4	7.5	25.1	2.95	0.59	0.63	0.22–1.84
Show insight	52.0	2.3	45.7	2.72	0.56	0.64	0–1.56
Empathy	50.4	1.7	47.9	2.66	0.55	0.62	0–1.56
Activity	50.4	3.7	45.9	2.95	0.74	0.84	0.22–2.41
**Self-reflection**^**c**^							
Amount of self-related thoughts	62.0	7.2	30.8	2.72	0.66	0.69	0.16–1.64

a Basic mood dimensions (MDMQ), item scale: 0–6.

b Performance judgments of the functional status, item scale: 1–6.

c Amount of self-related thoughts, item scale: 1–6.

*SOV, shares of variance indicated in percent, between patients (level 3), between days within a patient (level 2), between assessments within the days and a patient (level 1); M, mean over all participants; SD, standard deviation; rMSSD, root mean square of successive differences*.

### Procedure

On the day of the assessment, the principal investigator introduced the study procedure to the participants. During the assessment, each participant filled out the DESC and completed the neuropsychological tests. The assessment lasted ~1 h, depending on each participant's speed of performance. Thereafter, the patients were familiarized with the EMA, which included detailed instructions and examples of how the study questions should be answered by using the smartphone. The EMA was administered across 7 days, including a weekend. It started on the testing day just after the individual instructions had been given. Patients were asked the 18 questions eight times a day at semirandomized time points between 8 a.m. and 8 p.m., with a minimum of 60 min between each of two prompts. The participants were instructed that they could postpone answering the questions for 5, 10, or 15 min after a prompt had been given, in case answering immediately was not feasible (e.g., because of the patient's treatment). This option could be selected immediately after a prompt by touching a button on the smartphone screen. However, it was not possible to delay answering longer than 20 min after a prompt. This was to ensure that a situation-specific answer would be given in the randomly selected time period. The patient was prompted no more than five times for each assessment. The prompts were delivered auditory and by vibration via the smartphone.

### Data Analysis

Statistical analyses were conducted using the computing environment R version 3.5.1 (31) and with the packages *psych* (32), *lme4* (33), and *Hmisc* (34).

#### Compliance

Compliance was operationalized as the number of signal-contingent assessment that the participant answered and that was consistent with the experimental protocol. Each patient's compliance score was calculated as the relative proportion of completely and incompletely answered prompts to the number of prompts that were delivered during the entire study period. Compliance scores were calculated separately for weekdays and the weekend. The purpose was to determine whether compliance differed when patients had a relatively tight treatment schedule (i.e., on weekdays) and when they had more free time (i.e., on weekends). In addition, each patient's compliance was determined separately for each testing day, and a linear, mixed-effects model with random intercepts was calculated with each patient's compliance on the testing days as the dependent variable and the order of the testing days as the fixed-effects variable. The aim was to determine whether patients' compliance varied across the 7-day study period. Further, compliance scores were calculated for all patients across all of the testing days for each individual prompt (one to eight) to determine whether patients' compliance varied during the course of the day. Finally, these calculations were made: (a) the mean time in minutes between a prompt and the patient's first answer and (b) the mean response duration in minutes for all of the EMAs.

#### Fluctuations

To map response fluctuations, each patient's root mean square of successive differences (rMSSD) was calculated for the MDMQ, the judgments of performance, and the frequency of self-related thoughts since the last prompt. The rMSSD represents point-to-point variability in the time series (35, 36). Higher rMSSD values indicate greater fluctuation. In addition, for the same variables, the variances in the response behavior that was shared among the patients (level 3, between-person variability), among days within patients (level 2, within-person variability), and among individual assessments within days and patients (level 1, within-person variability) were calculated using simple (intercept only) three-level models. Responses to the respective variables were used as dependent variables. Additionally, the mean and SD were calculated for each variable.

#### Correlations Between the Patients' Compliance and Fluctuation and Their Level of Functioning

In order to identify relationships among the patient's compliance, fluctuation, and their level of functioning, a correlation matrix (using Pearson product-moment correlations) was constructed. Compliance rates and the mean rMSSD score for all investigated variables were correlated with the patient's age and functional level (i.e., DESC total score, VLMT learning score, mean ANT RT, and the total Barthel Index).

## Results

The results from the assessment for each patient are displayed in Table 1. At the time of admission, the patients had an average Barthel Index of 56 (SD = 20), which indicates moderately severe functional impairment. The relatively large SD in the patients' Barthel Index reflects the heterogeneity of the sample, which can also be seen in the neuropsychological test results (Table 1).

### Compliance

**Figure 1:**
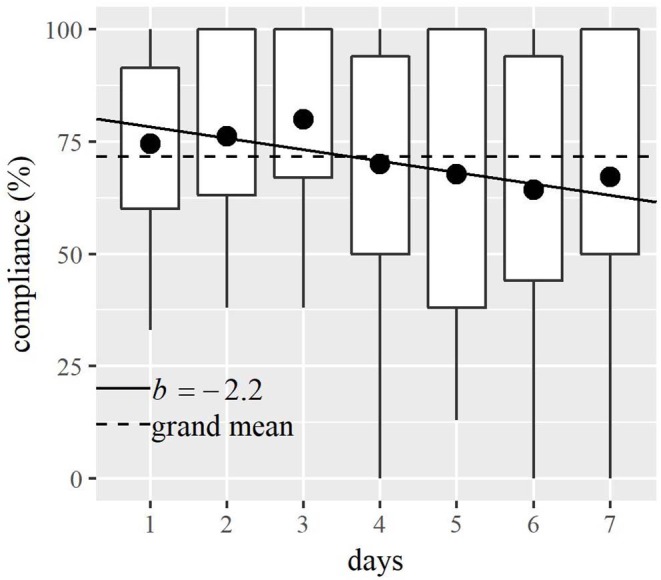
Relationship between patients' compliance (i.e., relative proportion of answered prompts to received prompts) and testing day. The dots show the mean compliance on each testing day. The lower and upper portions of the boxes correspond to the first and third quartiles. The lines leading the boxes indicate the range of compliance. The solid line shows the relationship between compliance and testing day. The broken line indicates that the mean compliance was 71.6% over the entire course of the study.

For the entire sample, a total of 779 random prompts were included in the study. Patients' mean compliance rate was 71.6% (SD = 18.3%, range = 46.2–100%; number of prompts: mean = 52, SD = 1.9, range = 46–54). Across all patients, only 1.8% of the prompts that were answered were incomplete; 4.7% were intentionally rejected; and 23.7% were ignored. During weekdays, the patients answered a mean of 71.1% of the received prompts (SD = 19.6%, range = 41.7–100%), and on weekends, they answered 71% of the received prompts (SD = 27.6%, range = 25–100%). The linear mixed-effects model with random intercepts to test the relationship between compliance on the individual testing days and the order of the testing days yielded a negative relationship (*b* = −2.2, SE = 1.16, *t*_(88)_ = −1.88, *p* = 0.06). This indicates that compliance decreased by 2.2% with each additional testing day. This result is shown in Figure 1. There was little variability in compliance within a single day (SD = 3.2%), but on average compliance was lowest on the first (68.3%) and last (68.2%) beep of the day. Patients responded on average 1.3 min after a prompt (SD = 2.9, range = 0.03–17.3 min), and they took about 1.5 min to answer the 18 questions (SD = 0.8, range = 0.4–7.4 min).

### Fluctuations

Across all variables, a mean of 55.1% of the variability in patients' responses was due to between-participant variability (SD = 10.5%, range = 33.1–74.7%, level 3), and 39.2% of the variance was explained by differences between the individual assessments within the days and a patient (SD = 10.4%, range = 22.4–61.4%, level 1). Approximately only 5.7% of the shared variance in patients' responses across all of the variables was explained by within-person differences between the testing days (SD = 3.7%, range = 1.7–15.1%, level 2). These results indicate that there was sufficient variability for examining both interindividual and intraindividual differences and for using multilevel modeling. The variance distribution for each variable and the respective mean rMSSD values are shown in Table 2. The rMSSD values indicate the point-to-point variability across time in relationship to the item scaling.

### Correlations Between Patients' Compliance and Fluctuation and Their Level of Functioning

There were no significant correlations (all *p* > 0.05) between patients' compliance or their mean fluctuation and their age or level of functioning (Table 3). However, to mention only one finding, there seems to be a tendency for less impaired patients, reflected in a higher Barthel Index, to have more fluctuation in their responses (*r* = 0.22, *p* > 0.05).

**Table 3:** Correlations between patients' compliance and response fluctuations and their age and level of functioning (*n* = 15).

	**Compliance**	**Mean fluctuation**
Age	0.35	−0.37
DESC	−0.06	−0.02
VLMT learning	−0.06	0.28
ANT RT	0.40	−0.07
Barthel Index	−0.03	0.22

None of the correlations reached significance. Mean fluctuation: mean rMSSD (root mean square of successive differences) across all variables.

*DESC, Rasch-based Depression Screening (n = 14); VLMT, Verbal Learning and Memory Test; ANT RT, mean response time (in milliseconds) in the Attention Network Test (n = 13)*.

## Discussion

The purpose of the current study was to replicate and extend the findings of previous feasibility studies of patients with an ABI. The patients in the present study had a relatively high compliance rate (71.6%), which is comparable to that found in previous studies of patients with an ABI (e.g., 9, 11, 13). This high rate was achieved, although (a) a high-frequency protocol was used (eight assessments per day across seven consecutive days), (b) the sample comprised patients with moderate ABIs, and (c) no incentive was offered for participating. In addition, only 1.8% of the prompts that patients responded to were incomplete, indicating that it was feasible for patients to answer 18 questions at this sampling frequency.

The results also indicate that patients' compliance did not differ between weekdays and weekend, and it varied little over the course of the day. Compliance, however, tended to decline by 2.2% with each successive day of testing. This decline across time did not occur in the study of Johnson et al. (8) investigating a stroke sample, who also analyzed the correlation of missing data and the duration of the study. Because the Johnson et al. (8) study included only five assessments per day across seven consecutive days, it would appear that a fatigue effect occurred in the present study caused by the higher sampling frequency. In addition, the different results in the two studies might be accounted for by differences in patient characteristics. At baseline, the stroke patients in the study of Johnson et al. (8) had a mean National Institutes of Health Stroke Scale score of 6.3 (SD = 4.1), which indicates minor to moderate stroke severity. In the present study, patients' mean Barthel Index indicated that they had at least moderate functional impairment, and 5 of the 15 patients had a hemiparesis.

The variance distributions of all variables that were investigated indicated sufficient variability, at both between-participant and within-participant levels, so that multilevel modeling could be used to explore both interindividual and intraindividual differences. This finding was informative because it had been unclear whether there would be sufficient fluctuation in the responses for some of the variables, such as participants' judgments of their performance (Table 2). The variance distributions indicated, in fact, that the judgments of performance varied both between and within participants over the entire course of the study. Interestingly, however, within-participant responses varied little between days, even though patient compliance tended to decrease over the course of the study. Another noteworthy finding was that neither patients' compliance nor their response variability was significantly related to their age or level of functioning. This suggests that EMA would be applicable for all patients, regardless of their age or the severity of their impairment. Accordingly, it would seem feasible to use EMA as a diagnostic tool regardless of the degree of patients' disability.

Certain limitations of the current study should be acknowledged. For instance, interpretation of the results is limited by the heterogeneity of the sample and its small size, which resulted in lower power, especially in the between-participant analyses. Accordingly, the impact of certain potentially confounding variables (e.g., cognitive impairment) cannot be completely ruled out despite the non-significant correlations. From a clinical point of view, an impaired awareness of one's illness or the presence of cognitive disorders would be expected to negatively affect participants' compliance and the validity of their answers. In addition, the preselection of patients and the inpatient setting in which testing occurred might have affected the results, and they limit the generalizability of the findings. The neuropsychologists who recruited the participants contacted only those patients who met the minimum requirements for participation and who they believed were suitable to participate. Accordingly, the investigator had contact with only preselected patients, not all of whom participated in the study. Spontaneous feedback from some of the patients suggested that they perceived their participation as annoying and burdensome. Finally, as in all EMA studies, we do not know the extent to which the patients' answers were genuine or whether they were merely being compliant. In any case, the fluctuations in patients' responses indicate that they were at least to a certain extent situation-specific.

Even if the results of the present study and the heterogeneity of the sample that was studied suggest that EMA would be suitable for patients with different levels of ABI impairment, it cannot be concluded that it would be feasible to use EMA with all patients with an ABI. In the present study, one-third of the sample suffered from a hemiparesis, and this did not seem to adversely affect their participation; nevertheless, other kinds of problems might place limitations on patients' participation in an EMA study. These include, for example, disorientation, severe sensory, motor, or cognitive impairments; aphasia; and apathy. The use of EMA might also be limited by other psychological factors such as social desirability and patients' individual differences.

Despite the aforementioned limitations, EMA appears to be a promising additional diagnostic tool for recording the temporal and situational course of patients' experiences and behaviors and the context in which they occur. We, therefore, see advantages of using EMA with patients with an ABI in both research and clinical settings. Additionally, smartphone applications that are designed for self-assessment might allow patients to assess and monitor their own symptoms, and the results could be shared with the treating clinician to adapt and improve the individual's treatment plan (11, 37, 38) and to assess the impact of the patient's illness on his or her family. This could be a first step toward meeting the need for more ecologically valid neuropsychological assessments (39).

## Data Availability Statement

The anonymized raw data on which this article is based will be available to any qualified researcher on request.

## Ethics Statement

The studies involving human participants were reviewed and approved by the Ethics Committee of the Medical Faculty of the RWTH Aachen University, Aachen, Germany. The patients provided their written informed consent to participate in this study.

## Author Contributions

SF, VM, and SG contributed to the conception and design of the study. AP and VV provided clinical support and assisted with participant recruitment. SF was responsible for the data collection and analysis and the graphical displays of the results. Finally, SF drafted the manuscript, and all of the co-authors critically reviewed it.

### Conflict of Interest

AP and VV were employed by VAMED Klinik Hattingen GmbH, Rehabilitation Centre for Neurology, Neurosurgery, Neuropaediatrics, Hattingen, Germany. The remaining authors declare that the research was conducted in the absence of any commercial or financial relationships that could be construed as a potential conflict of interest.
