# Supplementary material for: Ecological Momentary Assessment in Patients With an Acquired Brain Injury: A Pilot Study on Compliance and Fluctuations
Source: Front Neurol. 2020 Mar 5;11:115. doi: 10.3389/fneur.2020.00115 (PMC7066314; doi:10.3389/fneur.2020.00115)
Supplement: Supplementary file 1 [file Table_1.pdf]

Supplementary Material

Supplementary Table 1. Wording of the EMA questions in German and their translation into English.

EMA items	English	German
**Activity**		
What are you doing right now?	exercising	übe/trainiere
Multiple choice	therapy	Therapie
	drinking coffee	trinke Kaffee
	taking a break	ruhe mich aus
	watching TV	schaue TV
	eating	esse etwas
	listening to radio/music	höre Radio/Musik
	reading	lese
	surfing the Internet	surfe im Internet
	talking to others	rede mit anderen
	being outside	bin an der frischen Luft
**Social context**		
Who are you with right now?	on my own	mit niemandem
Multiple choice	with partner	mit Partner/Partnerin
	with relatives	mit Angehörigen
	with friends	mit Freunden

	with therapist	mit Therapeut
	with nurse(s)	mit Schwester/Pfleger
	with others	mit anderen Personen
**Mood***		
At the moment I feel …	**energetic arousal**	
Scale: *0 - 6*	tired – awake	müde – wach
	full of energy – without energy	energiegeladen – energielos
	**calmness**	
	agitated – calm	unruhig – ruhig
	relaxed – tense	entspannt – angespannt
	**valence**	
	content – discontent	zufrieden – unzufrieden
	unwell – well	unwohl – wohl
**Judgment of performance**		
What (school) grade would you give yourself since the last prompt for ...	memory	sich Sachen merken
	functional independence	Selbstständigkeit
Scale:	reliability	Zuverlässigkeit
1 (very good) – *6* (very bad)	self-confidence	Selbstbewusstsein
	learning	Lernfähigkeit
	understanding problems	Probleme verstehen

	show insight	Einsicht zeigen
	empathy	Einfühlungsvermögen
	activity	aktiv sein
**Self-reflection**		
Scale:<br><br>*1* (very little) – *6* (much)	How much have you been thinking about yourself since the last prompt?	Wie viel haben Sie seit der letzten Abfrage über sich selbst nachgedacht?
Note. * EMA adapted version of the Multidimensional Mood Questionnaire (1)		

1. Wilhelm P, Schoebi D. Assessing mood in daily life - Structural validity, sensitivity to change, and reliability of a short-scale to measure three basic dimensions of mood. *European Journal of Psychological Assessment* (2007) 23(4):258-67. doi: 10.1027/1015-5759.23.4.258.
